# Supplementary material for: Acyl carrier protein promotes MukBEF action in Escherichia coli chromosome organization-segregation
Source: Nat Commun. 2021 Nov 18;12:6721. doi: 10.1038/s41467-021-27107-9 (PMC8602292; doi:10.1038/s41467-021-27107-9)
Supplement: Supplementary file 1 — Supplementary Information [file 41467_2021_27107_MOESM1_ESM.pdf]

Supplementary Information

Acyl Carrier Protein promotes MukBEF action in *Escherichia coli* chromosome organization-segregation

Josh P. Prince[1], Jani R. Bolla[2, 3], Gemma L. M. Fisher[1], Jarno Mäkelä[1], Marjorie Fournier[1], Carol V. Robinson[2, 3], Lidia K. Arciszewska[1] and David J. Sherratt[1*]

[1] Department of Biochemistry, University of Oxford, South Parks Road, Oxford OX1 3QU, UK
[2] Physical and Theoretical Chemistry Laboratory, University of Oxford, South Parks Road, Oxford OX1 3QZ, UK
[3] The Kavli Institute for Nanoscience Discovery, South Parks Road, Oxford OX1 3QU, UK

* To whom correspondence should be addressed. Tel: +44 1865 613237; Fax: +44 1865 613213; Email: david.sherratt@bioch.ox.ac.uk

Present Address: Josh P. Prince: Meiosis Group, Medical Research Council London Institute of Medical Science, Du Cane Road, London W12 0NN, UK, Jani R. Bolla: Department of Plant Sciences, University of Oxford, South Parks Road, Oxford OX1 3QU, UK, Gemma L.M. Fisher, Cell Cycle Group, Medical Research Council London Institute of Medical Science, Du Cane Road, London W12 0NN, UK and Jarno Mäkelä, ChEM-H Institute, Stanford University, 290 Jane Stanford Way, CA 94305, US

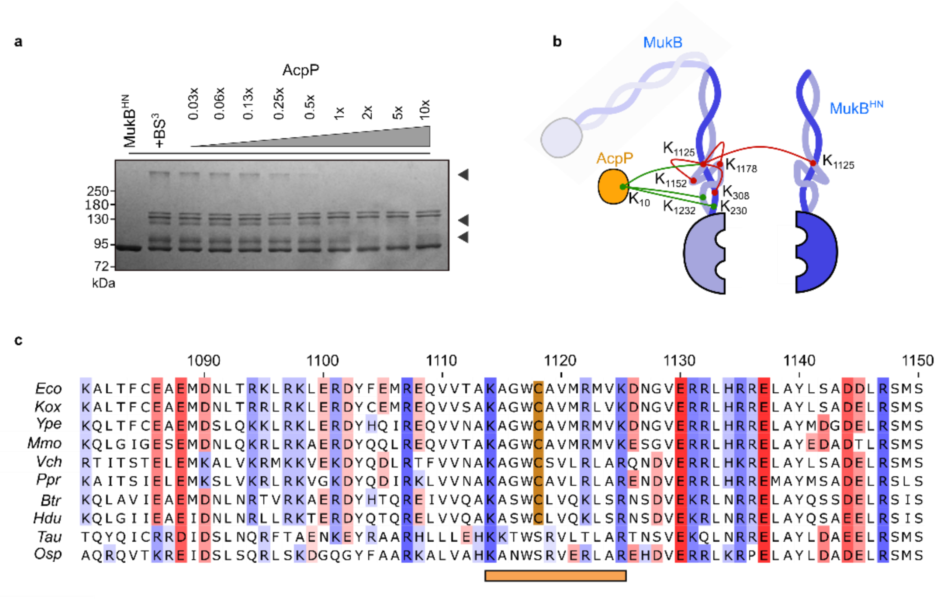

Supplementary Figure 1. Characterization of the MukB-AcpP interaction

(**a**) Identification of BS^3 cross-links, both inter- and intra-molecular cross-linking of $MukB^{HN}$ is inhibited by AcpP (indicated by grey arrows; top arrow, inter-molecular XLs; bottom arrows intra-molecular XLs). The gel is representative of at least two independent experiments. (**b**) Schematic of the cross-links in $MukB^{HN}$ that are inhibited by AcpP binding (red) and identified cross-links between AcpP and MukB (green). (**c**) Alignment of MukB sequences in the region bound by AcpP. Conservation in acidic and basic residues are indicated in red or blue respectively. The horizontal bar indicates the region analyzed in the work here. The conserved C1118 residue is also highlighted. Eco – *Escherichia coli*, Kox – *Klebsiella Oxytoca*, Ype – *Yersinia pestis*, Mmo – *Morganella morganii*, Vch – *Vibrio cholerae*, Ppr – *Photobacterium profundum*, Btr – *Bibersteinia trehalosi*, Hdu – *Haemophilus ducreyi*, Tau – *Tolumonas auensis*, Osp – *Oceanimonas sp*. Source data are provided as a Source Data file.

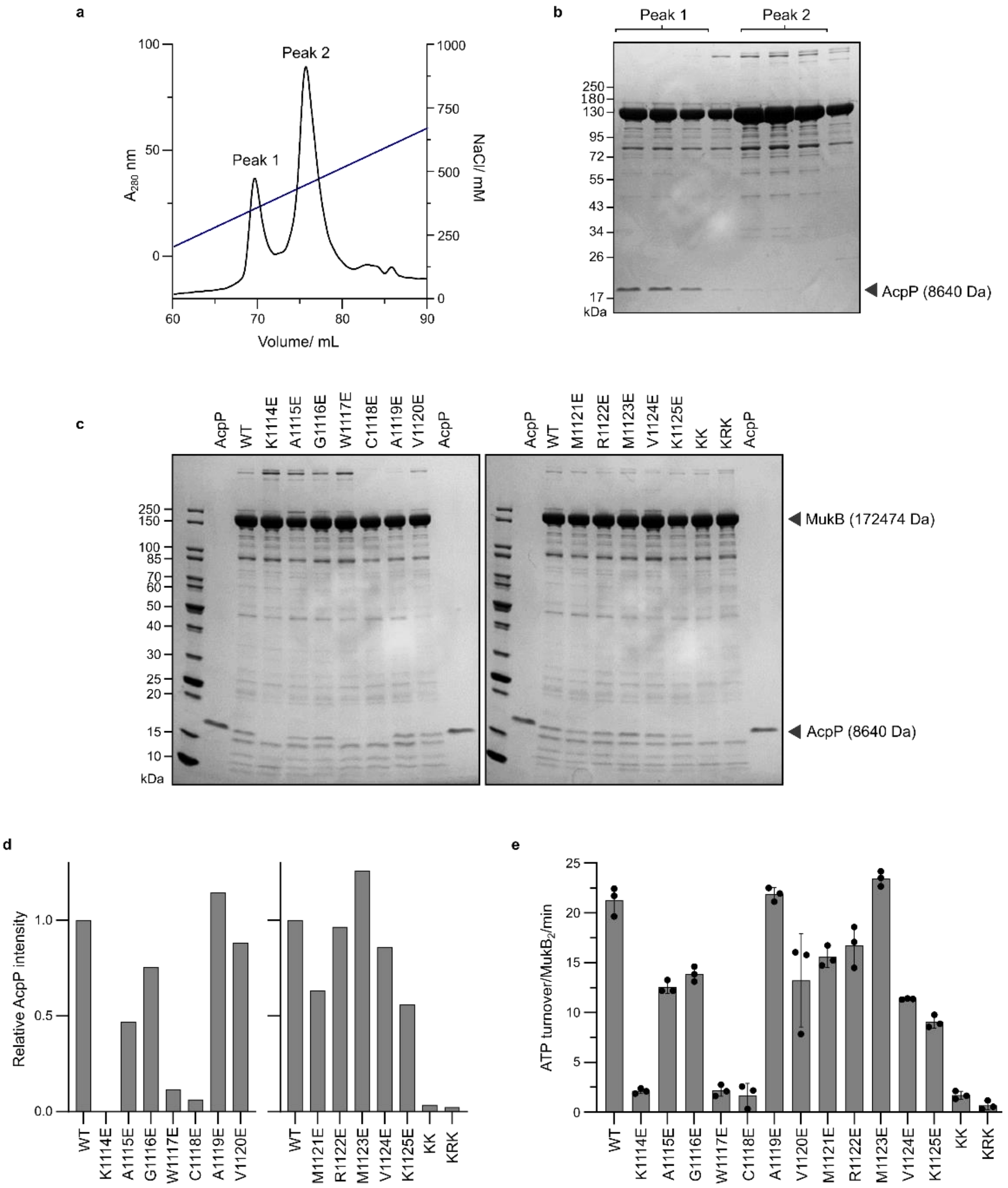

Supplementary Figure 2. Mutagenesis in the MukB-AcpP interface hinders AcpP co-purification and results in reduced ATPase activity

(**a**) Typical separation of MukB (peak 2) from MukB-AcpP (peak 1) using a salt gradient on a heparin column. (**b**) SDS-PAGE analysis of the peaks in (**a**). (**c**) SDS-PAGE analysis of TALON-purified MukB proteins, indicating the presence or absence of copurified AcpP. (**d**) Relative levels of AcpP associated with the indicated MukB proteins in (**c**), and (**e**) their ATPase levels (±SD from 3 technical repeats). The gels are representative of at least two independent experiments. Source data are provided as a Source Data file.

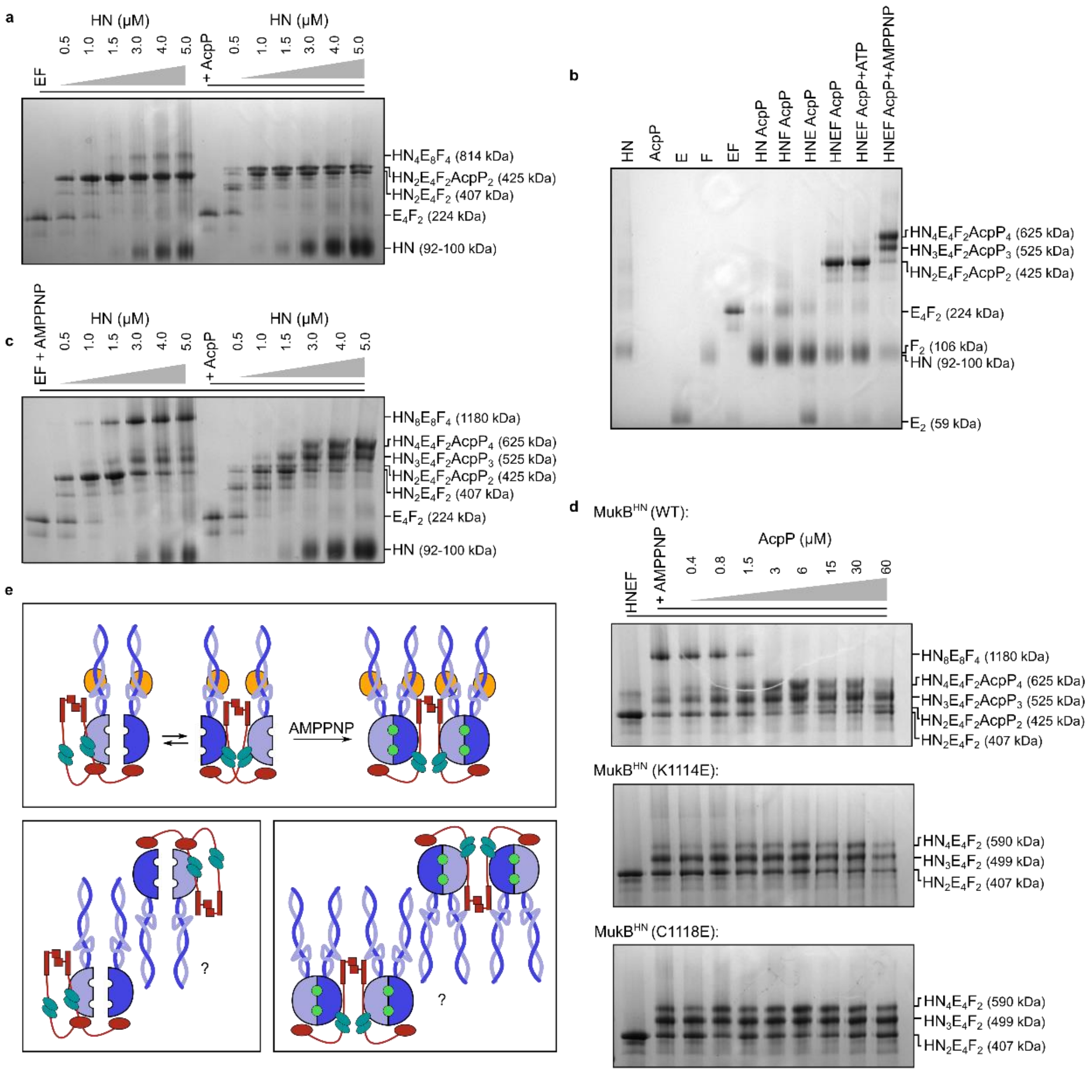

Supplementary Figure 3. AcpP prevents a coiled-coil interaction in $MukB^{HN}$

BN-PAGE analysis of complex formation in $MukB^{HN}$EF-AcpP. (**a**) AcpP hinders the formation of higher order $MukB^{HN}$EF complexes. (**b**) AMPPNP induces head engagement to form $MukB^{HN}{}_{3/4}E_4F_2$ complexes. (**c**) AcpP hinders the formation of AMPPNP-dependent higher order $MukB^{HN}$EF complexes (**d**) AcpP or mutagenesis in the MukB-AcpP interface hinders the formation of higher order complexes. (**e**) Schematic of nucleotide induced $MukB^{HN}$EF-AcpP head engagement (Top), or possible higher order complexes formed through coil-coil interactions in the absence of AcpP in head unengaged (Bottom, left) and head engaged (Bottom, right) complexes. The gels are representative of at least two independent experiments, theoretical masses are shown in parentheses.

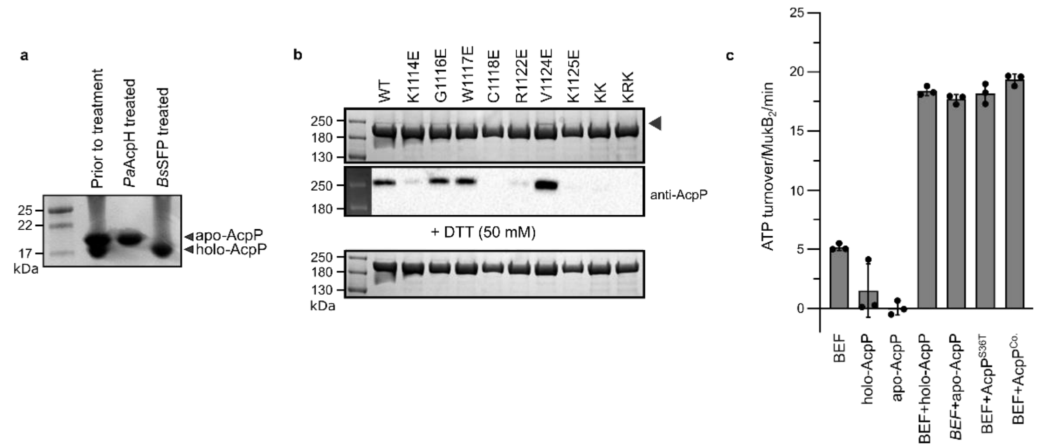

Supplementary Figure 4. Activities of apo-AcpP and holo-AcpP

(**a**) 20% urea-PAGE analysis of recombinant AcpP. Overexpression results in the production of apo- and holo-AcpP. (**b**) SDS-PAGE and western blot analysis of putative disulfide linked MukB-AcpP complexes (indicated by an arrow). (**c**) Initial ATPase activity measurements of MukB in the presence of various AcpP species (±SD from 3 technical repeats). For these experiments, AcpP that still contained the 6XHis-tag was used as its presence had no impact in the observed MukB ATPase activity. The gels are representative of at least two independent experiments. Source data are provided as a Source Data file.

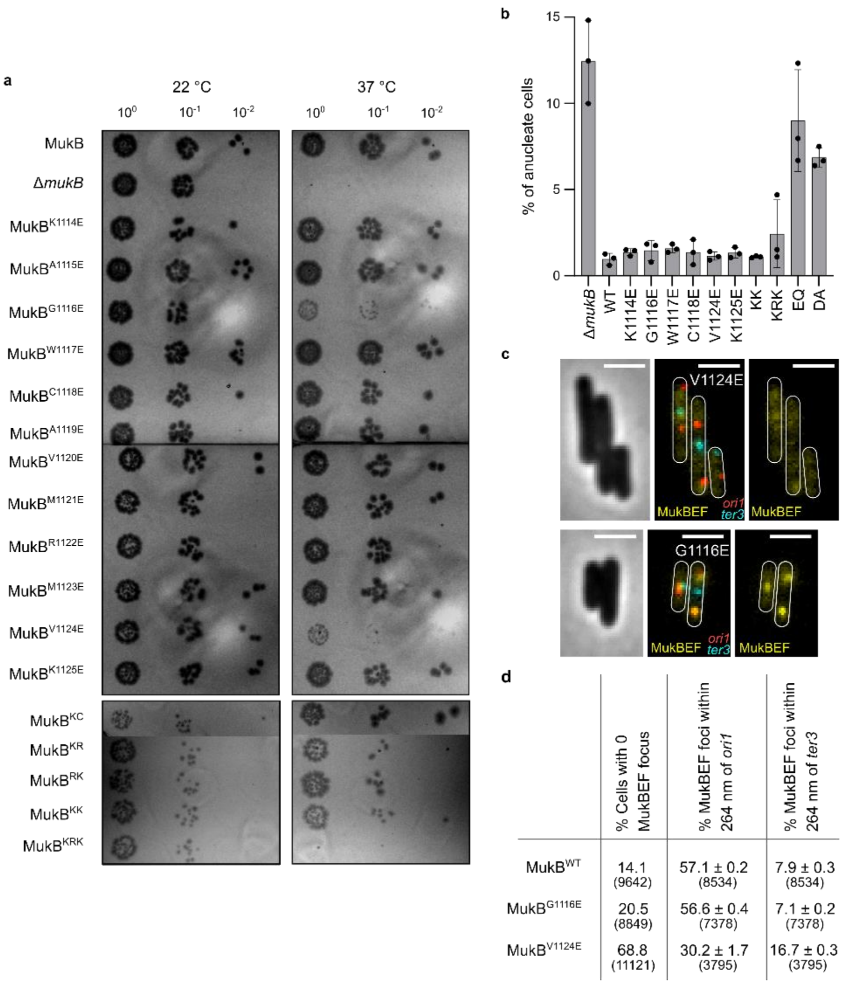

Supplementary Figure 5. Phenotypes of cells expressing MukB^{V1124E} and MukB^{G1116E} mutants

(**a**) Temperature sensitivity of WT and MukB mutant strains. Cells were grown in LB at 22 °C overnight, diluted and 20 µL spots of the dilutions plated and incubated as indicated. Images are representative of at least two independent experiments. (**b**) Percentage of anucleate cells within a given population (Δ*mukB* 33333 cells, WT 28459 cells, K1114E 25901 cells, G1116E 22242 cells, W1117E 14874 cells, C1118E 28585 cells, V1124E 35790 cells, K1125E 31013 cells, KK 20958 cells, KRK 19146 cells, EQ 22667 cells, DA 22272 cells; ± SD from three biological repeats). (**c**) Representative images of cells expressing MukB^{V1124E} and MukB^{G1116E} mutants (conditions as in Figure 4). Images are representative of cells from three biological repeats. Scale bars: 2 µm. (**d**) Analysis (as in Figure 4) of MukBEF foci in relation to *ori1* and *ter3* loci (± SD from 3 biological repeats. Number of cells analyzed in parentheses. Source data are provided as a Source Data file.

Sequence coverage for in-gel cross-linked samples

JP_003 / MukB^{HN}+AcpP+BS3_1

>DEF-USER-1_MukB^{HN}-His

MASIERGKFRSLTLINWNGFFARTFDLDELVTTLSGGNGAGKSTTMAAFVTALIPDLTLLHFRNTTEAGATSGSRDKGLHGKLKA
GVCYSMLDTINSRHQRVVVGVRLQQVAGRDRKVDIKPFAIQGLPMSVQPTQLVTETLNERQARVLPLNELKDKLEAMEGVQFK
QFNSITDYHSLMFDLGIIARRLRSASDRSKFYRLIEASLYGGISSAITRSLRDYLLPENSGVRKAFQDMEAALRENRMTLEAIRVTQ
SDRDLFKHLISEATNYVAADYMRHANERRVHLDKALEFRRELHTSRQQLAAEQYKHVDMARELAEHNGAEGDLEADYQAASDH
LNLVADPGGGSEGGEFSEGGSGSGAEERARIRRDELHAQLSNNRSRRNQLEKALTFCEAEMDNLTRKLRKLERDYFEMREQV
VTAKAGWCAVMRMVKDNGVERRLHRRELAYLSADDLRSMSDKALGALRLAVADNEHLRDVLRMSEDPKRPERKIQFFVAVYQ
HLRERIRQDIIRTDDPVEAIEQMEIELSRLTEELTSREQKLAISSRSVANIIRKTIQREQNRIRMLNQGLQNVSFGQVNSVRLNVNV
RETHAMLLDVLSEQHEQHQDLFNSNRLTFSEALAKLYQRLNPQIDMGQRTPQTIGEELLDYRNYLEMEVEVNRGSDGWLRAES
GALSTGEAIGTGMSILVMVVQSWEDESRRLRGKDISPCRLLFLDEAARLDARSIATLFELCERLQMQLIIAAPENISPEKGTTYKLV
RKVFQNTEHVHVVGLRGFAPLPETLPGTDEAPSQASAAALEHHHHHH

JP_004 / MukB^{HN}+AcpP+BS3_2

>DEF-USER-1_MukB^{HN}-His

MASIERGKFRSLTLINWNGFFARTFDLDELVTTLSGGNGAGKSTTMAAFVTALIPDLTLLHFRNTTEAGATSGSRDKGLHGKLKA
GVCYSMLDTINSRHQRVVVGVRLQQVAGRDRKVDIKPFAIQGLPMSVQPTQLVTETLNERQARVLPLNELKDKLEAMEGVQFK
QFNSITDYHSLMFDLGIIARRLRSASDRSKFYRLIEASLYGGISSAITRSLRDYLLPENSGVRKAFQDMEAALRENRMTLEAIRVTQ
SDRDLFKHLISEATNYVAADYMRHANERRVHLDKALEFRRELHTSRQQLAAEQYKHVDMARELAEHNGAEGDLEADYQAASDH
LNLVADPGGGSEGGEFSEGGSGSGAEERARIRRDELHAQLSNNRSRRNQLEKALTFCEAEMDNLTRKLRKLERDYFEMREQV
VTAKAGWCAVMRMVKDNGVERRLHRRELAYLSADDLRSMSDKALGALRLAVADNEHLRDVLRMSEDPKRPERKIQFFVAVYQ
HLRERIRQDIIRTDDPVEAIEQMEIELSRLTEELTSREQKLAISSRSVANIIRKTIQREQNRIRMLNQGLQNVSFGQVNSVRLNVNV
RETHAMLLDVLSEQHEQHQDLFNSNRLTFSEALAKLYQRLNPQIDMGQRTPQTIGEELLDYRNYLEMEVEVNRGSDGWLRAES
GALSTGEAIGTGMSILVMVVQSWEDESRRLRGKDISPCRLLFLDEAARLDARSIATLFELCERLQMQLIIAAPENISPEKGTTYKLV
RKVFQNTEHVHVVGLRGFAPLPETLPGTDEAPSQASAAALEHHHHHH

JP_005 / MukB^{HN}+AcpP+BS3_3

>DEF-USER-1_MukB^{HN}-His

MASIERGKFRSLTLINWNGFFARTFDLDELVTTLSGGNGAGKSTTMAAFVTALIPDLTLLHFRNTTEAGATSGSRDKGLHGKLKA
GVCYSMLDTINSRHQRVVVGVRLQQVAGRDRKVDIKPFAIQGLPMSVQPTQLVTETLNERQARVLPLNELKDKLEAMEGVQFK
QFNSITDYHSLMFDLGIIARRLRSASDRSKFYRLIEASLYGGISSAITRSLRDYLLPENSGVRKAFQDMEAALRENRMTLEAIRVTQ
SDRDLFKHLISEATNYVAADYMRHANERRVHLDKALEFRRELHTSRQQLAAEQYKHVDMARELAEHNGAEGDLEADYQAASDH
LNLVADPGGGSEGGEFSEGGSGSGAEERARIRRDELHAQLSNNRSRRNQLEKALTFCEAEMDNLTRKLRKLERDYFEMREQV
VTAKAGWCAVMRMVKDNGVERRLHRRELAYLSADDLRSMSDKALGALRLAVADNEHLRDVLRMSEDPKRPERKIQFFVAVYQ
HLRERIRQDIIRTDDPVEAIEQMEIELSRLTEELTSREQKLAISSRSVANIIRKTIQREQNRIRMLNQGLQNVSFGQVNSVRLNVNV
RETHAMLLDVLSEQHEQHQDLFNSNRLTFSEALAKLYQRLNPQIDMGQRTPQTIGEELLDYRNYLEMEVEVNRGSDGWLRAES
GALSTGEAIGTGMSILVMVVQSWEDESRRLRGKDISPCRLLFLDEAARLDARSIATLFELCERLQMQLIIAAPENISPEKGTTYKLV
RKVFQNTEHVHVVGLRGFAPLPETLPGTDEAPSQASAAALEHHHHHH

JP_006 / MukB^{HN}+BS3_1

>DEF-USER-1_MukB^{HN}-His

MASIERGKFRSLTLINWNGFFARTFDLDELVTTLSGGNGAGKSTTMAAFVTALIPDLTLLHFRNTTEAGATSGSRDKGLHGKLKA
GVCYSMLDTINSRHQRVVVGVRLQQVAGRDRKVDIKPFAIQGLPMSVQPTQLVTETLNERQARVLPLNELKDKLEAMEGVQFK
QFNSITDYHSLMFDLGIIARRLRSASDRSKFYRLIEASLYGGISSAITRSLRDYLLPENSGVRKAFQDMEAALRENRMTLEAIRVTQ
SDRDLFKHLISEATNYVAADYMRHANERRVHLDKALEFRRELHTSRQQLAAEQYKHVDMARELAEHNGAEGDLEADYQAASDH
LNLVADPGGGSEGGEFSEGGSGSGAEERARIRRDELHAQLSNNRSRRNQLEKALTFCEAEMDNLTRKLRKLERDYFEMREQV
VTAKAGWCAVMRMVKDNGVERRLHRRELAYLSADDLRSMSDKALGALRLAVADNEHLRDVLRMSEDPKRPERKIQFFVAVYQ
HLRERIRQDIIRTDDPVEAIEQMEIELSRLTEELTSREQKLAISSRSVANIIRKTIQREQNRIRMLNQGLQNVSFGQVNSVRLNVNV
RETHAMLLDVLSEQHEQHQDLFNSNRLTFSEALAKLYQRLNPQIDMGQRTPQTIGEELLDYRNYLEMEVEVNRGSDGWLRAES
GALSTGEAIGTGMSILVMVVQSWEDESRRLRGKDISPCRLLFLDEAARLDARSIATLFELCERLQMQLIIAAPENISPEKGTTYKLV
RKVFQNTEHVHVVGLRGFAPLPETLPGTDEAPSQASAAALEHHHHHH

JP_007 / MukB^{HN}+BS3_2

>DEF-USER-1_MukB^{HN}-His

MASIERGKFRSLTLINWNGFFARTFDLDELVTTLSGGNGAGKSTTMAAFVTALIPDLTLLHFRNTTEAGATSGSRDKGLHGKLKA
GVCYSMLDTINSRHQRVVVGVRLQQVAGRDRKVDIKPFAIQGLPMSVQPTQLVTETLNERQARVLPLNELKDKLEAMEGVQFK
QFNSITDYHSLMFDLGIIARRLRSASDRSKFYRLIEASLYGGISSAITRSLRDYLLPENSGVRKAFQDMEAALRENRMTLEAIRVTQ
SDRDLFKHLISEATNYVAADYMRHANERRVHLDKALEFRRELHTSRQQLAAEQYKHVDMARELAEHNGAEGDLEADYQAASDH
LNLVADPGGGSEGGEFSEGGSGSGAEERARIRRDELHAQLSNNRSRRNQLEKALTFCEAEMDNLTRKLRKLERDYFEMREQV
VTAKAGWCAVMRMVKDNGVERRLHRRELAYLSADDLRSMSDKALGALRLAVADNEHLRDVLRMSEDPKRPERKIQFFVAVYQ
HLRERIRQDIIRTDDPVEAIEQMEIELSRLTEELTSREQKLAISSRSVANIIRKTIQREQNRIRMLNQGLQNVSFGQVNSVRLNVNV
RETHAMLLDVLSEQHEQHQDLFNSNRLTFSEALAKLYQRLNPQIDMGQRTPQTIGEELLDYRNYLEMEVEVNRGSDGWLRAES
GALSTGEAIGTGMSILVMVVQSWEDESRRLRGKDISPCRLLFLDEAARLDARSIATLFELCERLQMQLIIAAPENISPEKGTTYKLV
RKVFQNTEHVHVVGLRGFAPLPETLPGTDEAPSQASAAALEHHHHHH

JP_008 / MukB^{HN}+BS3_3

>DEF-USER-1_MukB^{HN}-His

MASIERGKFRSLTLINWNGFFARTFDLDELVTTLSGGNGAGKSTTMAAFVTALIPDLTLLHFRNTTEAGATSGSRDKGLHGKLKA
GVCYSMLDTINSRHQRVVVGVRLQQVAGRDRKVDIKPFAIQGLPMSVQPTQLVTETLNERQARVLPLNELKDKLEAMEGVQFK
QFNSITDYHSLMFDLGIIARRLRSASDRSKFYRLIEASLYGGISSAITRSLRDYLLPENSGVRKAFQDMEAALRENRMTLEAIRVTQ
SDRDLFKHLISEATNYVAADYMRHANERRVHLDKALEFRRELHTSRQQLAAEQYKHVDMARELAEHNGAEGDLEADYQAASDH
LNLVADPGGGSEGGEFSEGGSGSGAEERARIRRDELHAQLSNNRSRRNQLEKALTFCEAEMDNLTRKLRKLERDYFEMREQV
VTAKAGWCAVMRMVKDNGVERRLHRRELAYLSADDLRSMSDKALGALRLAVADNEHLRDVLRMSEDPKRPERKIQFFVAVYQ
HLRERIRQDIIRTDDPVEAIEQMEIELSRLTEELTSREQKLAISSRSVANIIRKTIQREQNRIRMLNQGLQNVSFGQVNSVRLNVNV
RETHAMLLDVLSEQHEQHQDLFNSNRLTFSEALAKLYQRLNPQIDMGQRTPQTIGEELLDYRNYLEMEVEVNRGSDGWLRAES
GALSTGEAIGTGMSILVMVVQSWEDESRRLRGKDISPCRLLFLDEAARLDARSIATLFELCERLQMQLIIAAPENISPEKGTTYKLV
RKVFQNTEHVHVVGLRGFAPLPETLPGTDEAPSQASAAALEHHHHHH

JP_047 / MukB^{HN}+BS3_4

>DEF-USER-1_MukB^{HN}-His

MASIERGKFRSLTLINWNGFFARTFDLDELVTTLSGGNGAGKSTTMAAFVTALIPDLTLLHFRNTTEAGATSGSRDKGLHGKLKA
GVCYSMLDTINSRHQRVVVGVRLQQVAGRDRKVDIKPFAIQGLPMSVQPTQLVTETLNERQARVLPLNELKDKLEAMEGVQFK
QFNSITDYHSLMFDLGIIARRLRSASDRSKFYRLIEASLYGGISSAITRSLRDYLLPENSGVRKAFQDMEAALRENRMTLEAIRVTQ
SDRDLFKHLISEATNYVAADYMRHANERRVHLDKALEFRRELHTSRQQLAAEQYKHVDMARELAEHNGAEGDLEADYQAASDH
LNLVADPGGGSEGGEFSEGGSGSGAEERARIRRDELHAQLSNNRSRRNQLEKALTFCEAEMDNLTRKLRKLERDYFEMREQV
VTAKAGWCAVMRMVKDNGVERRLHRRELAYLSADDLRSMSDKALGALRLAVADNEHLRDVLRMSEDPKRPERKIQFFVAVYQ
HLRERIRQDIIRTDDPVEAIEQMEIELSRLTEELTSREQKLAISSRSVANIIRKTIQREQNRIRMLNQGLQNVSFGQVNSVRLNVNV
RETHAMLLDVLSEQHEQHQDLFNSNRLTFSEALAKLYQRLNPQIDMGQRTPQTIGEELLDYRNYLEMEVEVNRGSDGWLRAES
GALSTGEAIGTGMSILVMVVQSWEDESRRLRGKDISPCRLLFLDEAARLDARSIATLFELCERLQMQLIIAAPENISPEKGTTYKLV
RKVFQNTEHVHVVGLRGFAPLPETLPGTDEAPSQASAAALEHHHHHH

JP_048 / MukB^{HN}+BS3_5

>DEF-USER-1_MukB^{HN}-His

MASIERGKFRSLTLINWNGFFARTFDLDELVTTLSGGNGAGKSTTMAAFVTALIPDLTLLHFRNTTEAGATSGSRDKGLHGKLKA
GVCYSMLDTINSRHQRVVVGVRLQQVAGRDRKVDIKPFAIQGLPMSVQPTQLVTETLNERQARVLPLNELKDKLEAMEGVQFK
QFNSITDYHSLMFDLGIIARRLRSASDRSKFYRLIEASLYGGISSAITRSLRDYLLPENSGVRKAFQDMEAALRENRMTLEAIRVTQ
SDRDLFKHLISEATNYVAADYMRHANERRVHLDKALEFRRELHTSRQQLAAEQYKHVDMARELAEHNGAEGDLEADYQAASDH
LNLVADPGGGSEGGEFSEGGSGSGAEERARIRRDELHAQLSNNRSRRNQLEKALTFCEAEMDNLTRKLRKLERDYFEMREQV
VTAKAGWCAVMRMVKDNGVERRLHRRELAYLSADDLRSMSDKALGALRLAVADNEHLRDVLRMSEDPKRPERKIQFFVAVYQ
HLRERIRQDIIRTDDPVEAIEQMEIELSRLTEELTSREQKLAISSRSVANIIRKTIQREQNRIRMLNQGLQNVSFGQVNSVRLNVNV
RETHAMLLDVLSEQHEQHQDLFNSNRLTFSEALAKLYQRLNPQIDMGQRTPQTIGEELLDYRNYLEMEVEVNRGSDGWLRAES
GALSTGEAIGTGMSILVMVVQSWEDESRRLRGKDISPCRLLFLDEAARLDARSIATLFELCERLQMQLIIAAPENISPEKGTTYKLV
RKVFQNTEHVHVVGLRGFAPLPETLPGTDEAPSQASAAALEHHHHHH

Sequence coverage for in-solution cross-linked samples

JP_054 / MukBEF+AcpP+BS3

>DEF-USER1_MukB-His

MASIERGKFRSLTLINWNGFFARTFDLDELVTTLSGGNGAGKSTTMAAFVTALIPDLTLLHFRNTTEAGATSGSRDKGLHGKLKA
GVCYSMLDTINSRHQRVVVGVRLQQVAGRDRKVDIKPFAIQGLPMSVQPTQLVTETLNERQARVLPLNELKDKLEAMEGVQFK
QFNSITDYHSLMFDLGIIARRLRSASDRSKFYRLIEASLYGGISSAITRSLRDYLLPENSGVRKAFQDMEAALRENRMTLEAIRVTQ
SDRDLFKHLISEATNYVAADYMRHANERRVHLDKALEFRRELHTSRQQLAAEQYKHVDMARELAEHNGAEGDLEADYQAASDH
LNLVQTALRQQEKIERYEADLDELQIRLEEQNEVVAEAIERQQENEARAEAAELEVDELKSQLADYQQALDVQQTRAIQYNQAIA
ALNRAKELCHLPDLTADCAAEWLETFQAKELEATEKMLSLEQKMSMAQTAHSQFEQAYQLVVAINGPLARNEAWDVARELLRE
GVDQRHLAEQVQPLRMRLSELEQRLREQQEAERLLADFCKRQGKNFDIDELEALHQELEARIASLSDSVSNAREERMALRQEQ
EQLQSRIQSLMQRAPVWLAAQNSLNQLSEQCGEEFTSSQDVTEYLQQLLEREREAIVERDEVGARKNAVDEEIERLSQPGGSE
DQRLNALAERFGGVLLSEIYDDVSLEDAPYFSALYGPSRHAIVVPDLSQVTEHLEGLTDCPEDLYLIEGDPQSFDDSVFSVDELE
KAVVVKIADRQWRYSRFPEVPLFGRAARESRIESLHAEREVLSERFATLSFDVQKTQRLHQAFSRFIGSHLAVAFESDPEAEIRQ
LNSRRVELERALSNHENDNQQQRIQFEQAKEGVTALNRILPRLNLLADDSLADRVDEIRERLDEAQEAARFVQQFGNQLAKLEPI
VSVLQSDPEQFEQLKEDYAYSQQMQRDARQQAFALTEVVQRRAHFSYSDSAEMLSGNSDLNEKLRERLEQAEAERTRAREAL
RGHAAQLSQYNQVLASLKSSYDTKKELLNDLQRELQDIGVRADSGAEERARIRRDELHQLSNNRSRRNQLEKALTFCEAEMDN
LTRKLRKLERDYFEMREQVVTAKAGWCAVMRMVKDNGVERRLHRRELAYSADDLRSMSDKALGALRLAVADNEHLRDVLRMS
EDPKRPERKIQFFVAVYQHLRERIRQDIIRTDDPVEAIEQMEIELSRLTEELTSREQKLAISSRSVANIIRKTIQREQNRIRMLNQGL
QNVSFGQVNSVRLNVNVRETHAMLLDVLSEQHEQHQDLFNSNRLTFSEALAKLYQRLNPQIDMGQRTPQTIGEELLDYRNYLE
MEVEVNRGSDGWLRAESGALSTGEAIGTGMSILVMVVQSWEDESRRLRGKDISPCRLLFLDEAARLDARSIATLFELCERLQMQ
LIIAAPENISPEKGTTYKLVRVFQNTEHVHVVGLRGFAPQLPETLPGTDEAPSQASADPNSSSVDKLAAALEHHHHHH

>DEF-USER2_His-AcpP

MGSSHHHHHHSSGLVPRGSHMMSTIEERVKKIIGEQLGVKQEEVTNNASFVEDLGADSLDTVELVMALEEEFDTEIPDEEAEKIT
TVQAAIDYINGHQA

>DEF_USER3_MukE-His

MMSSTNIEQVMPVKLAQALANPLFPALDSALRSGRHIGLDELDNHAFLMDFQEYLEEFYARYNVELIRAPEGFFYLRPRSTTLIPR
SVLSELDMMVGKILCYLYLSPERLANEGIFTQQELYDELLTLADEAKLLKLVNNRSTGSDVDRQKLQEKVRSSLNRLRRLGMVWF
MGHDSSKFRITESVFRFGADVRAGDDPREAQRRLIRDGEAMPIENHLQLNDETEENQPDSGEEEADPNSSSVDKLAAALEHHH
HHH

>DEF-USER4_His-MukF

MGSSHHHHHHSSGLVPRGSHMASSEFSQTVPELVAWARKNDFSISLPVDRLSFLLAVATLNGERLDGEMSEGELVDAFRHVSD
AFEQTSETIGVRANNAINDMVRQRLLNRFTSEQAEGNAIYRLTPLGIGITDYYIRQREFSTLRLSMQLSIVAGELKRAADAAEEGG
DEFHWHRNVYAPLKYSVAEIFDSIDLTQRLMDEQQQQVKDDIAQLLNKDWRAAISSCELLLSETSGTLRELQDTLEAAGDKLQAN
LLRIQDATMTHDDLHFVDRLVFDLQSKLDRIISWGQQSIDLWIGYDRHVHKFIRTAIDMDKNRVFAQRLRQSVQTYFDEPWALTY
ANADRLLDMRDEEMALRDEEVTGELPEDLEYEEFNEIREQLAAIIEEQLAVYKTRQVPLDLGLVVREYLSQYPRARHFDVARIVID
QAVRLGVAQADFTGLPAKWQPINDYGAKVQAHVIDKY

JP_055 / MukBEF+BS3

>DEF-USER1_MukB-His

MASIERGKFRSLTLINWNGFFARTFDLDELVTTLSGGNGAGKSTTMAAFVTALIPDLTLLHFRNTTEAGATSGSRDKGLHGKLKA
GVCYSMLDTINSRHQRVVVGVRLQQVAGRDRKVDIKPFAIQGLPMSVQPTQLVTETLNERQARVLPLNELKDKLEAMEGVQFK
QFNSITDYHSLMFDLGIIARRLRSASDRSKFYRLIEASLYGGISSAITRSLRDYLLPENSGVRKAFQDMEAALRENRMTLEAIRVTQ
SDRDLFKHLISEATNYVAADYMRHANERRVHLDKALEFRRELHTSRQQLAAEQYKHVDMARELAEHNGAEGDLEADYQAASDH
LNLVQTALRQQEKIERYEADLDELQIRLEEQNEVVAEAIERQQENEARAEAAELEVDELKSQLADYQQALDVQQTRAIQYNQAIA
ALNRAKELCHLPDLTADCAAEWLETFQAKELEATEKMLSLEQKMSMAQTAHSQFEQAYQLVVAINGPLARNEAWDVARELLRE
GVDQRHLAEQVQPLRMRLSELEQRLREQQEAERLLADFCKRQGKNFDIDELEALHQELEARIASLSDSVSNAREERMALRQEQ
EQLQSRIQSLMQRAPVWLAAQNSLNQLSEQCGEEFTSSQDVTEYLQQLLEREREAIVERDEVGARKNAVDEEIERLSQPGGSE
DQRLNALAERFGGVLLSEIYDDVSLEDAPYFSALYGPSRHAIVVPDLSQVTEHLEGLTDCPEDLYLIEGDPQSFDDSVFSVDELE
KAVVVKIADRQWRYSRFPEVPLFGRAARESRIESLHAEREVLSERFATLSFDVQKTQRLHQAFSRFIGSHLAVAFESDPEAEIRQ
LNSRRVELERALSNHENDNQQQRIQFEQAKEGVTALNRILPRLNLLADDSLADRVDEIRERLDEAQEAARFVQQFGNQLAKLEPI
VSVLQSDPEQFEQLKEDYAYSQQMQRDARQQAFALTEVVQRRAHFSYSDSAEMLSGNSDLNEKLRERLEQAEAERTRAREAL
RGHAAQLSQYNQVLASLKSSYDTKKELLNDLQRELQDIGVRADSGAEERARIRRDELHQLSNNRSRRNQLEKALTFCEAEMDN
LTRKLRKLERDYFEMREQVVTAKAGWCAVMRMVKDNGVERRLHRRELAYLSADDLRSMSDKALGALRLAVADNEHLRDVLRM
SEDPKRPERKIQFFVAVYQHLRERIRQDIIRTDDPVEAIEQMEIELSRLTEELTSREQKLAISSRSVANIIRKTIQREQNRIRMLNQG
LQNVSFGQVNSVRLNVNVRETHAMLLDVLSEQHQHQDLFNSNRLTFSEALAKLYQRLNPQIDMGQRTPQTIGEELLDYRNYLE
MEVEVNRGSDGWLRAESGALSTGEAIGTGMSILVMVVQSWEDESRRLRGKDISPCRLLFLDEAARLDARSIATLFELCERLQMQ
LIIAAPENISPEKGTTYKLVRKVFQNTEHVHVVGLRGFAPQLPETLPGTDEAPSQASADPNSSSVDKLAAALEHHHHHH

>DEF-USER2_His-AcpP

MGSSHHHHHHSSGLVPRGSHMMSTIEERVKKIIGEQLGVKQEEVTNNASFVEDLGADSLDTVELVMALEEEFDTEIPDEEAEKIT
TVQAAIDYINGHQA

>DEF_USER3_MukE-His

MMSSTNIEQVMPVKLAQALANPLFPALDSALRSGRHIGLDELDNHAFLMDFQEYLEEFYARYNVELIRAPEGFFYRPRSTTLIPRS
VLSELDMMVGKILCYLYLSPERLANEGIFTQQELYDELLTLADEAKLLKLVNNRSTGSDVDRQKLQEKVRSSLNRLRRLGMVWF
MGHDSSKFRITESVFRFGADVRAGDDPREAQRRLIRDGEAMPIENHLQLNDETEENQPDSGEEEADPNSSSVDKLAAALEHHH
HHH

>DEF-USER4_His-MukF

MGSSHHHHHHSSGLVPRGSHMASSEFSQTVPELVAWARKNDFSISLPVDRLSFLLAVATLNGERLDGEMSEGELDAFRHVSDA
FEQTSETIGVRANNAINDMVRQRLLNRFTSEQAEGNAIYRLTPLGIGITDYYIRQREFSTLRLSMQLSIVAGELKRAADAAEEGGD
EFHWHRNVYAPLKYSVAEIFDSIDLTQRLMDEQQQQVKDDIAQLLNKDWRAAISSCELLLSETSGTLRELQDTLEAAGDKLQANL
LRIQDATMTHDDLHFVDRLVFDLQSKLDRIISWGQQSIDLWIGYDRHVHKFIRTAIDMDKNRVFAQRLRQSVQTYFDEPWALTYA
NADRLLDMRDEEMALRDEEVTGELPEDLEYEEFNEIREQLAAIIEEQLAVYKTRQVPLDLGLVVREYLSQYPRARHFDVARIVIDQ
AVRLGVAQADFTGLPAKWQPINDYGAKVQAHVIDKY

Supplementary Figure 6. Protein sequence coverage

Protein sequence coverage (in yellow) and cross-linked peptides (in purple) identified by LC-MS/MS analysis of in-gel and in-solution digested samples containing MukB^{HN} ± AcpP and MukBEF ± AcpP proteins respectively.

a

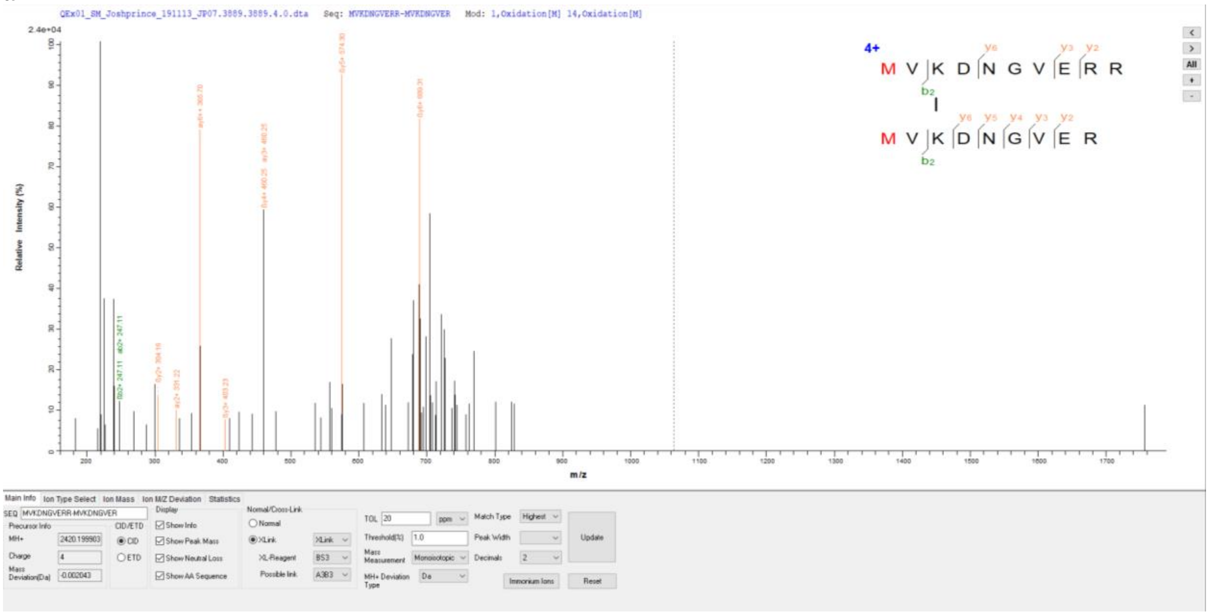
QEx01_SM_Joshprince_191113_JP07.3889.3889.4.0.dta Seq: MVKDNGVERR-MVKDNGVER Mod: 1,Oxidation[M] 14,Oxidation[M]
2.4e+04
Relative Intensity (%)
m/z
4+
M V K D N G V E R R
M V K D N G V E R
Main Info
Ion Type Select
Ion Mass
Ion M/Z Deviation
Statistics
SEQ MVKDNGVERR-MVKDNGVER
Precursor Info
MH+ 2420.199903
Charge 4
Mass Deviation(Da) -0.002043
CID/ETD
CID
ETD
Display
Show Info
Show Peak Mass
Show Neutral Loss
Show AA Sequence
Normal/Cross-Link
Normal
XLink
XL-Reagent BS3
Possible link A3B3
TOL 20 ppm
Threshold(%) 1.0
Mass Measurement Monoisotopic
MH+ Deviation Type Da
Match Type Highest
Peak Width
Decimals 2
Update
Immonium Ions
Reset

b

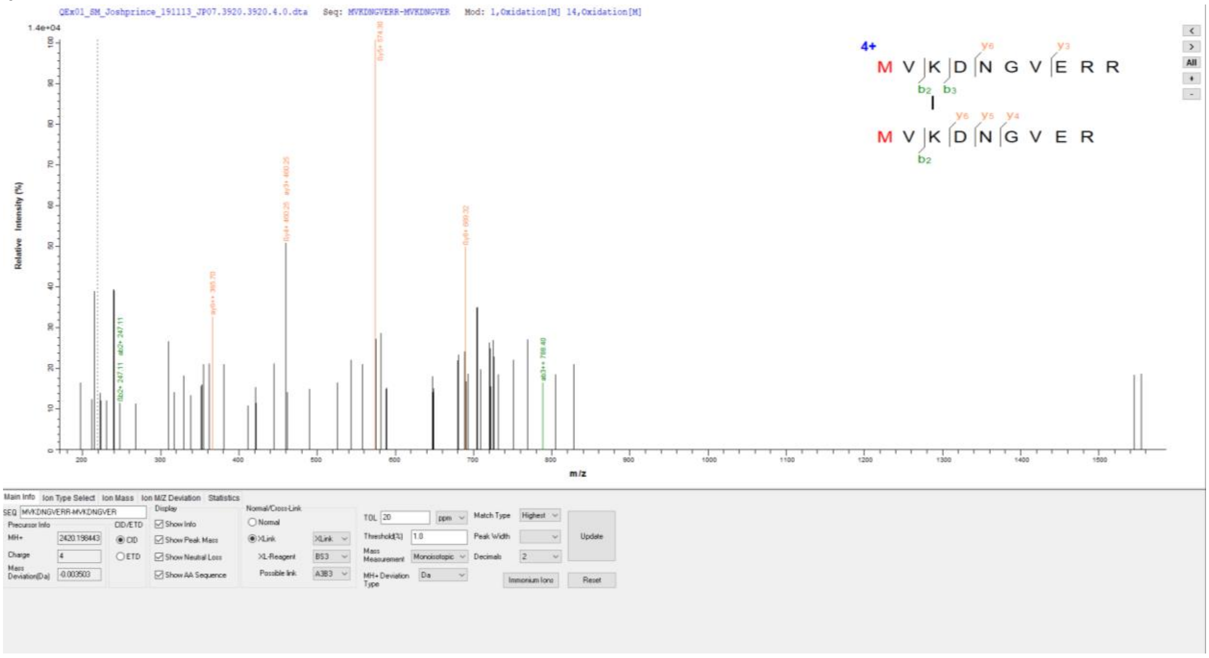
QEx01_SM_Joshprince_191113_JP07.3920.3920.4.0.dta Seq: MVKDNGVERR-MVKDNGVER Mod: 1,Oxidation[M] 14,Oxidation[M]
1.4e+04
Relative Intensity (%)
m/z
4+
M V K D N G V E R R
M V K D N G V E R
Main Info
Ion Type Select
Ion Mass
Ion M/Z Deviation
Statistics
SEQ MVKDNGVERR-MVKDNGVER
Precursor Info
MH+ 2420.198443
Charge 4
Mass Deviation(Da) -0.003503
CID/ETD
CID
ETD
Display
Show Info
Show Peak Mass
Show Neutral Loss
Show AA Sequence
Normal/Cross-Link
Normal
XLink
XL-Reagent BS3
Possible link A3B3
TOL 20 ppm
Threshold(%) 1.0
Mass Measurement Monoisotopic
MH+ Deviation Type Da
Match Type Highest
Peak Width
Decimals 2
Update
Immonium Ions
Reset

c

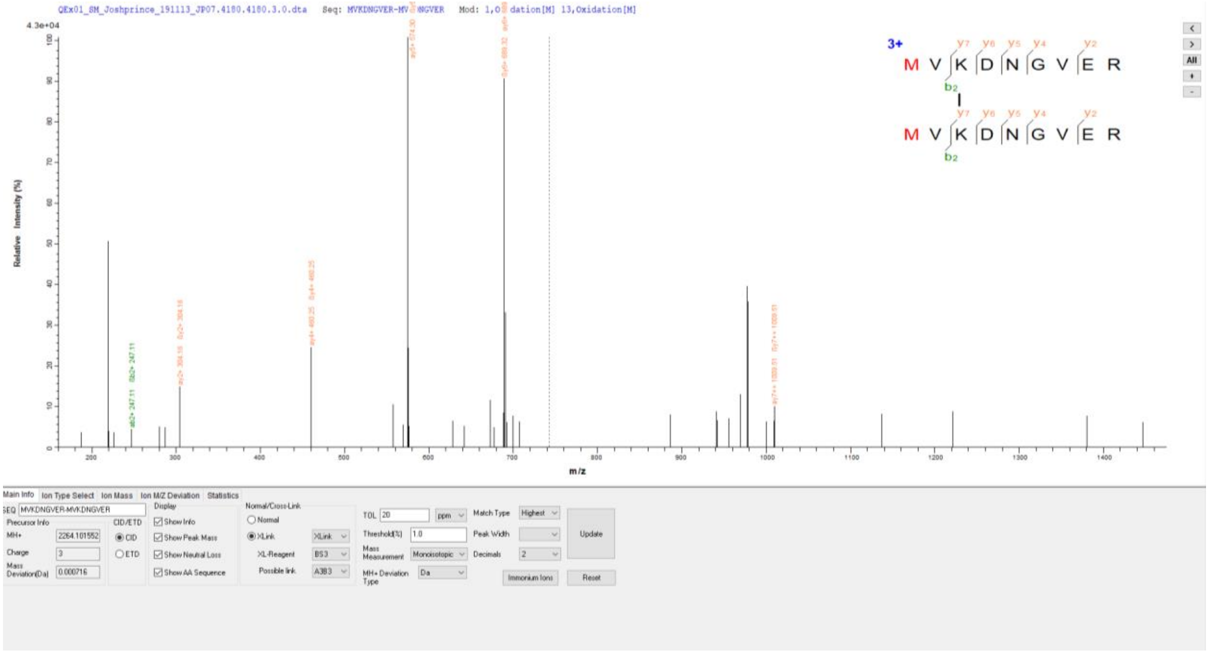
3+
M V K D N G V E R
m/z
Relative Intensity (%)

d

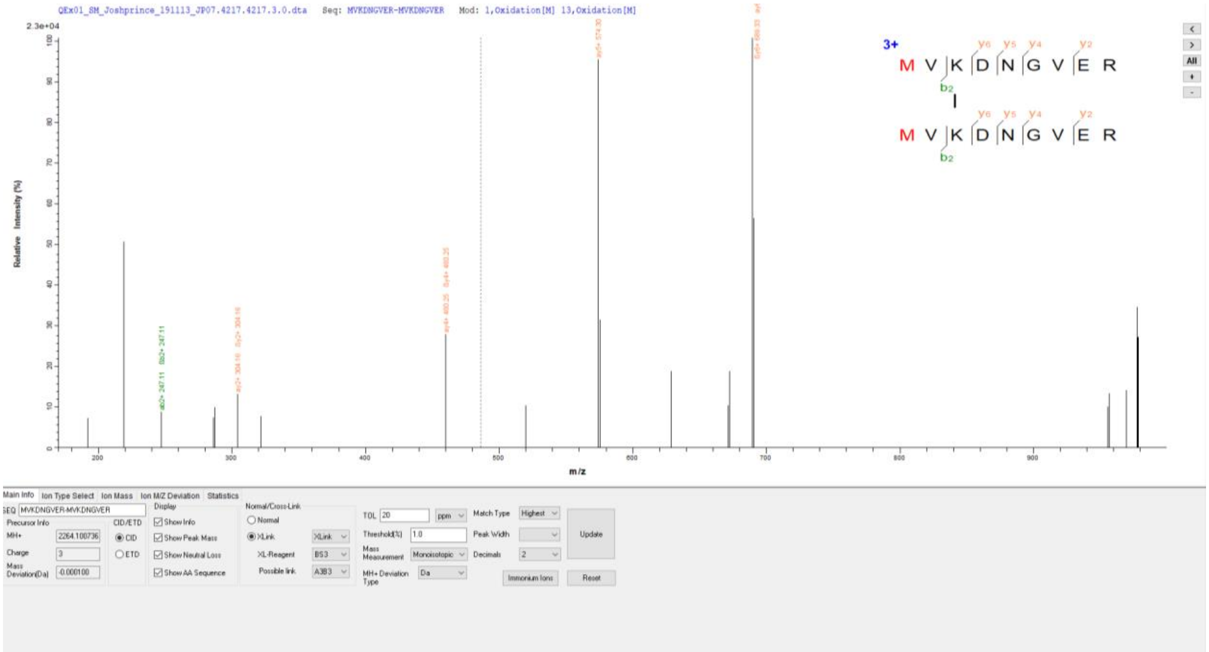
Seq: MVKDNGVER-MVKDNGVER
3+
M V K D N G V E R
m/z
Relative Intensity (%)

e

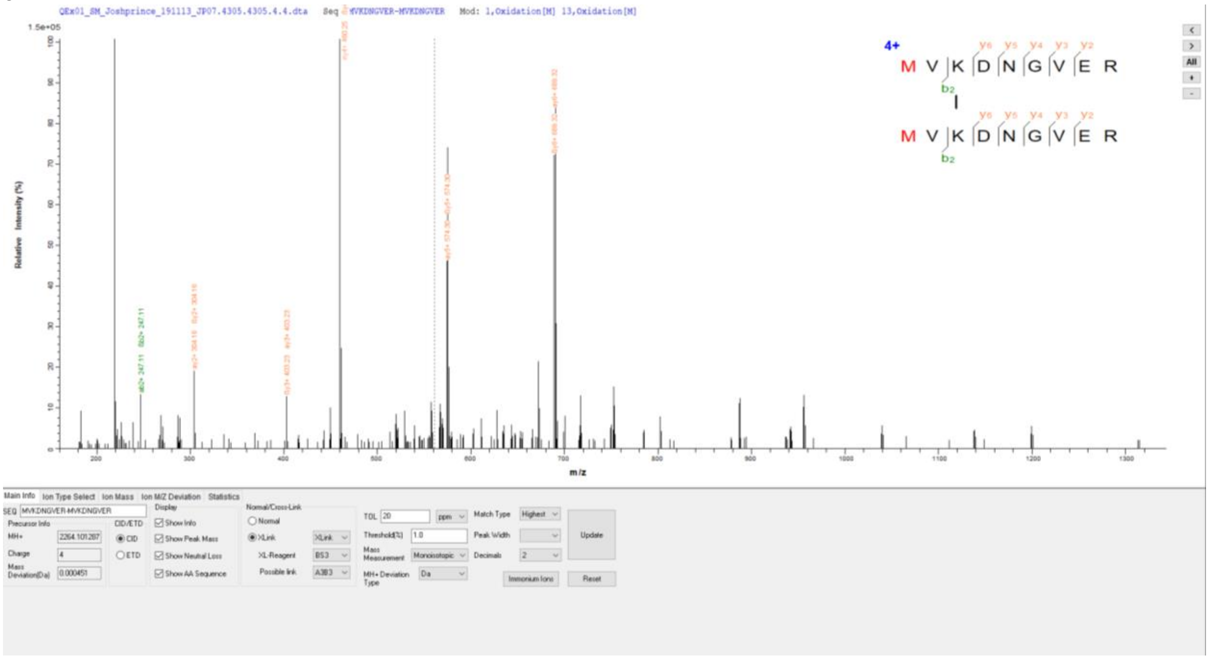
QEx01_SM_Joshprince_191113_JP07.4305.4305.4.4.dta
Seq MVKDNGVER-MVKDNGVER
Mod: 1,Oxidation[M] 13,Oxidation[M]
Relative Intensity (%)
m/z
4+
M V K D N G V E R
M V K D N G V E R
Main Info
Ion Type Select
Ion Mass
Ion M/Z Deviation
Statistics
Update
Reset

f

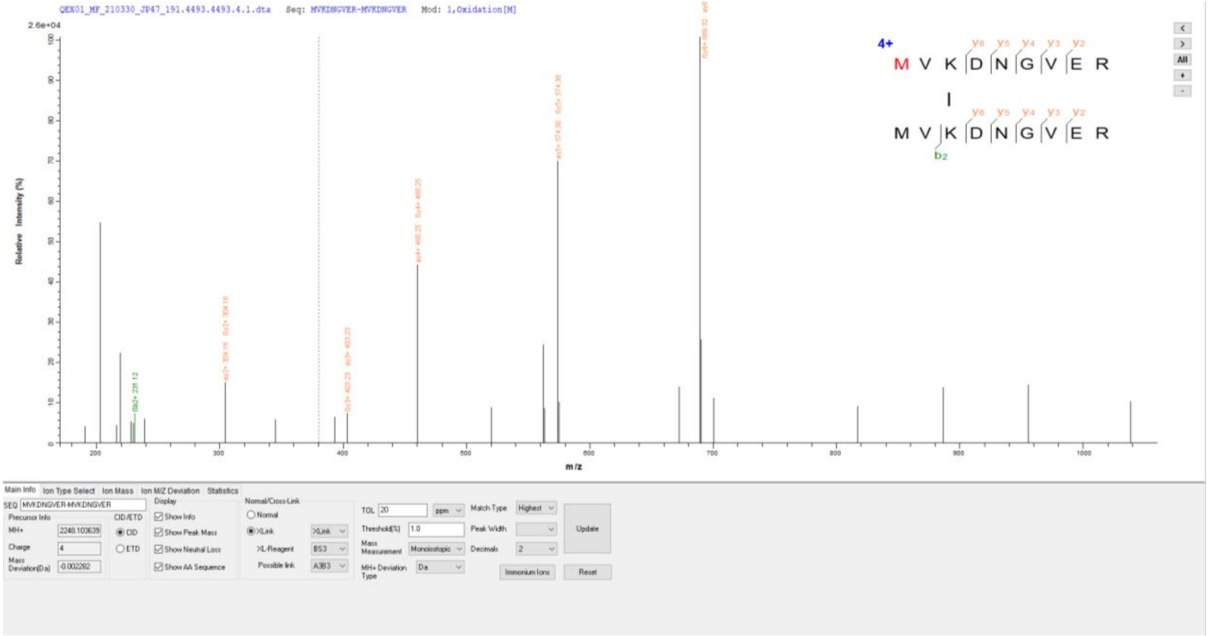
QEX01_MF_210330_JP47_191.4493.4493.4.1.dta
Seq: MVKDNGVER-MVKDNGVER
Mod: 1,Oxidation[M]
Relative Intensity (%)
m/z
4+
M V K D N G V E R
M V K D N G V E R
Main Info
Ion Type Select
Ion Mass
Ion M/Z Deviation
Statistics
Update
Reset

g

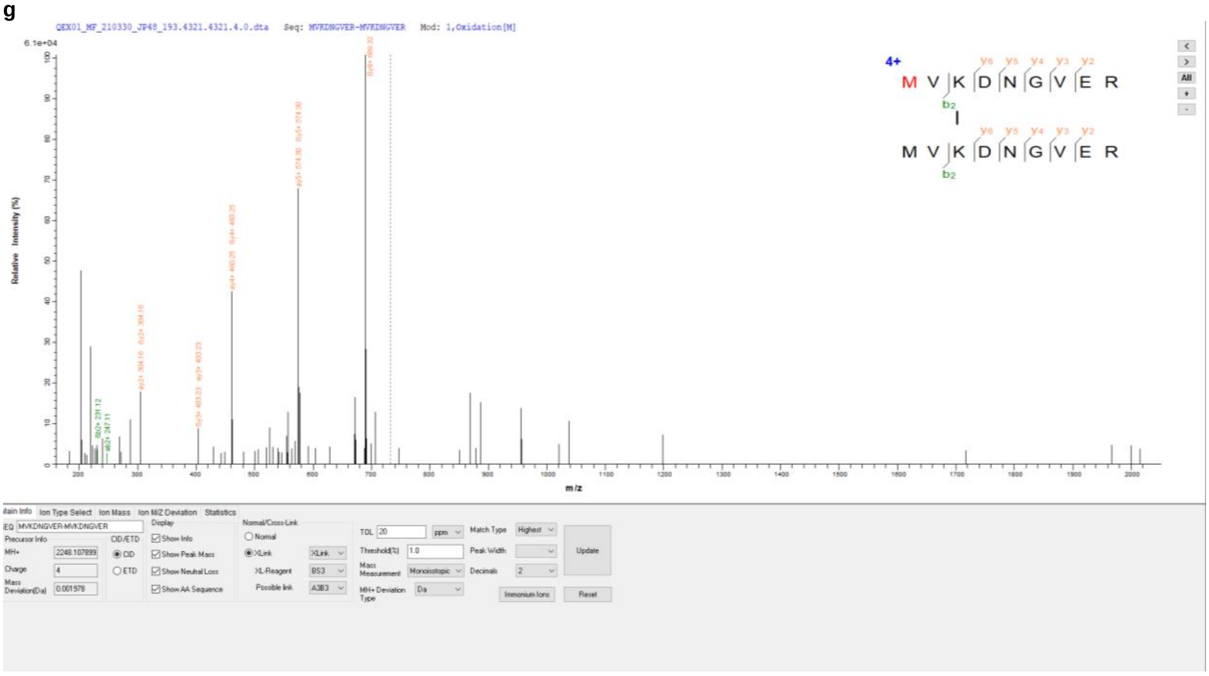
QEX01_MF_210330_JP48_193.4321.4321.4.0.dta Seq: MVKDNGVER-MVKDNGVER Mod: 1,Oxidation[M]
4+
M V K D N G V E R
M V K D N G V E R
Relative Intensity (%)
m/z
Main Info
Ion Type Select
Ion Mass
Ion M/Z Deviation
Statistics
Precursor Info
MH+
2248.107899
Charge
4
Mass Deviation(Da)
0.001578
CID/ETD
Display
Show Info
Show Peak Mass
Show Neutral Loss
Show AA Sequence
Normal/Cross-Link
Normal
XLink
XL-Reagent
BS3
Possible link
TOL
20
ppm
Threshold(%)
1.0
Mass Measurement
Monoisotopic
MH+ Deviation Type
Da
Match Type
Highest
Peak Width
Decimals
2
Update
Immonium Ions
Reset

h

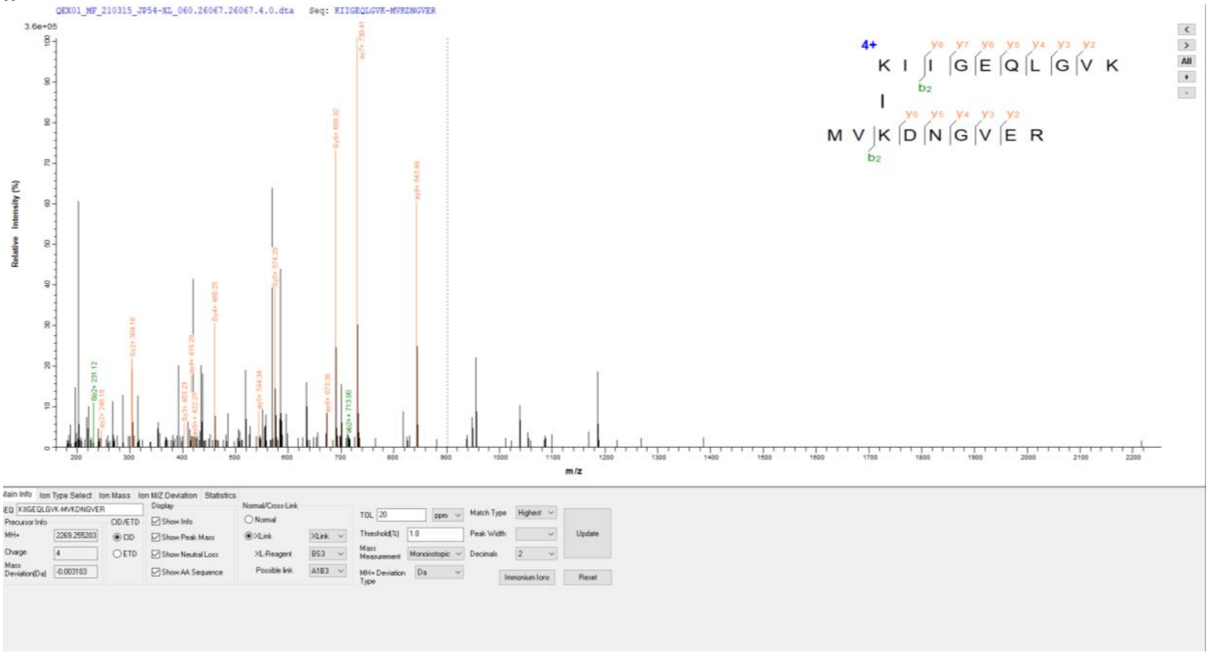
QEX01_MF_210315_JP54-XL_060.26067.26067.4.0.dta Seq: KIIGEQLGVK-MVKDNGVER
4+
K I I G E Q L G V K
M V K D N G V E R
Relative Intensity (%)
m/z
Main Info
Ion Type Select
Ion Mass
Ion M/Z Deviation
Statistics
KIIGEQLGVK-MVKDNGVER
Precursor Info
MH+
2269.255203
Charge
4
Mass Deviation(Da)
-0.003103
CID/ETD
Display
Show Info
Show Peak Mass
Show Neutral Loss
Show AA Sequence
Normal/Cross-Link
Normal
XLink
XL-Reagent
BS3
Possible link
TOL
20
ppm
Threshold(%)
1.0
Mass Measurement
Monoisotopic
MH+ Deviation Type
Da
Match Type
Highest
Peak Width
Decimals
2
Update
Immonium Ions
Reset

i

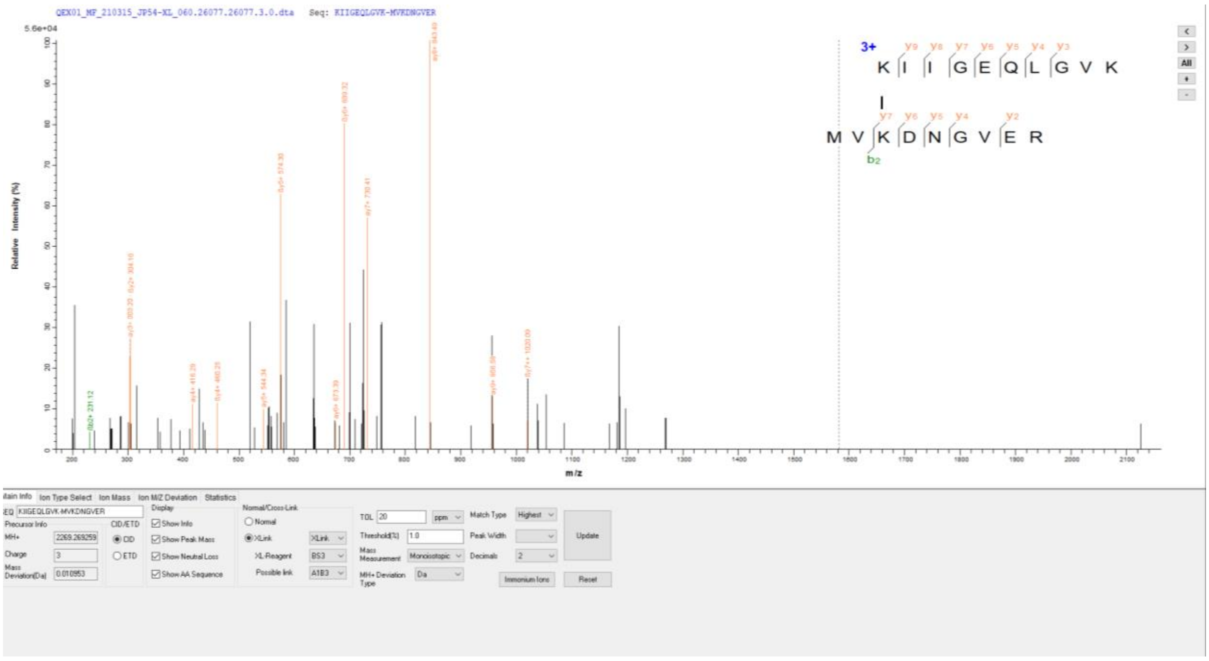
QEX01_MF_210315_JP54-XL_060.26077.26077.3.0.dta Seq: KIIGEQLGVK-MVKDNGVER
Relative Intensity (%)
m/z
3+
K I I G E Q L G V K
M V K D N G V E R
MH+ 2269.269259
Charge 3
Mass Deviation(Da) 0.010953

j

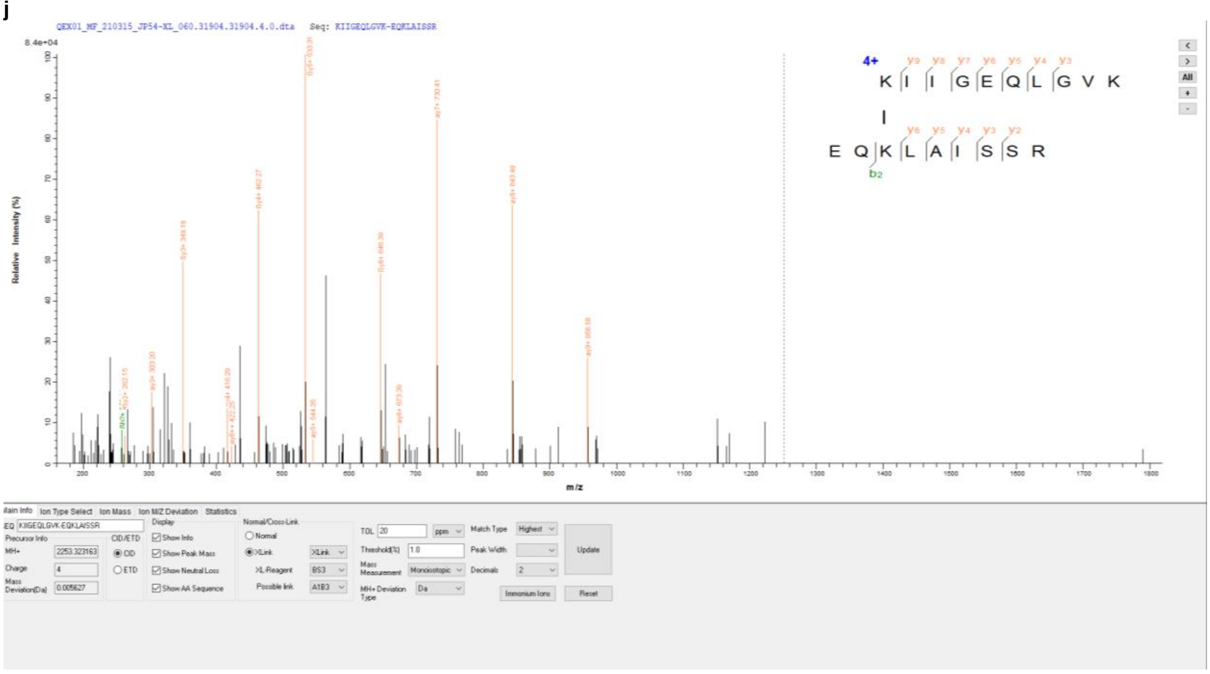
QEX01_MF_210315_JP54-XL_060.31904.31904.4.0.dta Seq: KIIGEQLGVK-EQKLAISSR
Relative Intensity (%)
m/z
4+
K I I G E Q L G V K
E Q K L A I S S R
MH+ 2253.323163
Charge 4
Mass Deviation(Da) 0.005627

k

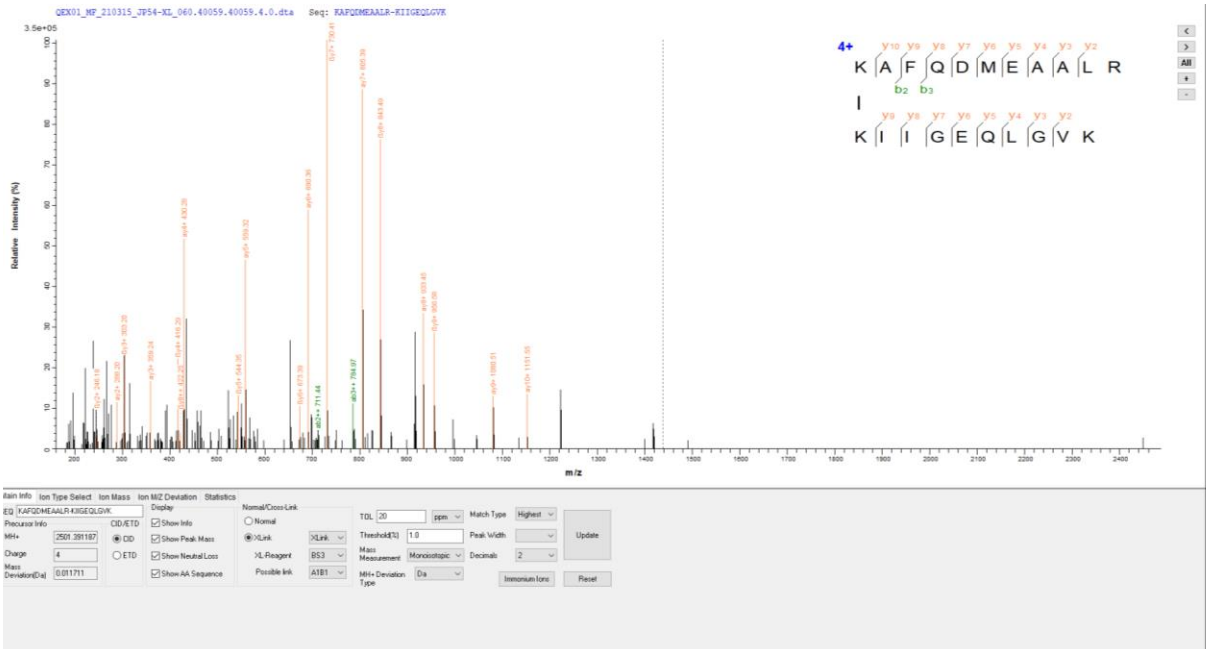

l

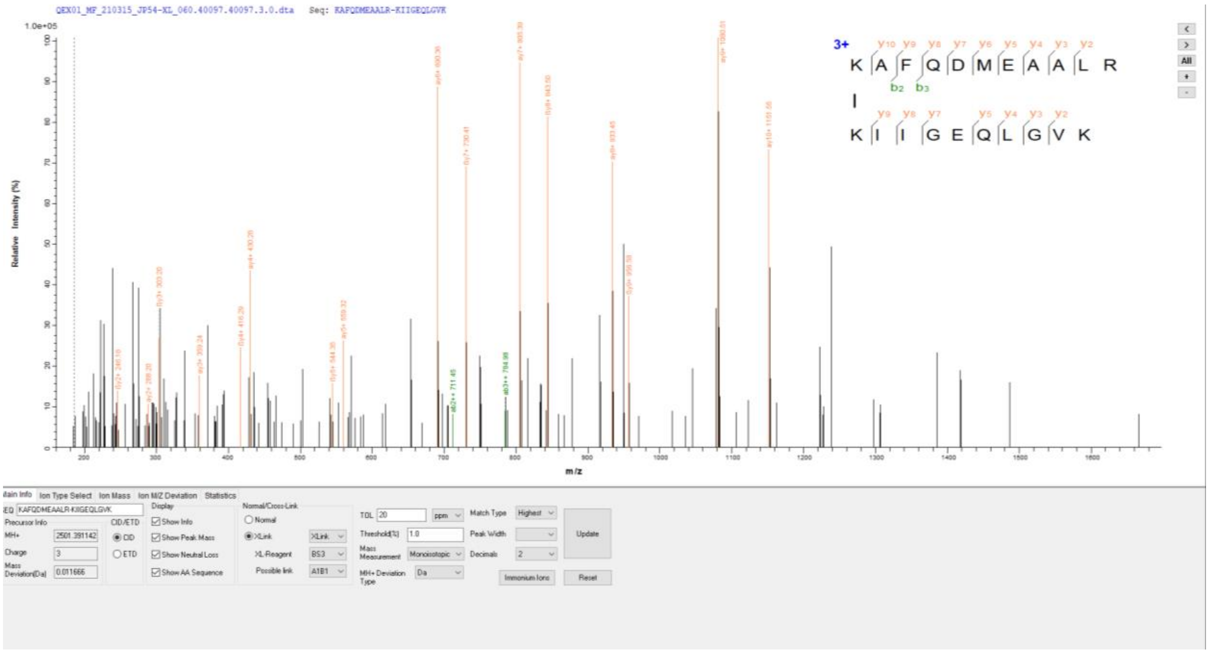

Supplementary Figure 7. Example MS/MS spectra of identified cross-links

MS/MS spectra of the intermolecular K1125-K1125 cross-link identified in samples containing MukB^{HN} and BS^{3}; JP007 (**a**-**e**), JP047 (**f**) and JP048 (**g**). MS/MS spectra of identified cross-links between residue K10 of AcpP and K1125 (**h** and **i**), K1232 (**j**) and K230 (**k**-**l**) of MukB.
