# Supplementary material for: Acyl carrier protein promotes MukBEF action in Escherichia coli chromosome organization-segregation
Source: Nat Commun. 2021 Nov 18;12:6721. doi: 10.1038/s41467-021-27107-9 (PMC8602292; doi:10.1038/s41467-021-27107-9)
Supplement: Supplementary file 3 — Description of Additional Supplementary Files [file 41467_2021_27107_MOESM3_ESM.pdf]

Description of Additional Supplementary Files

File Name: Supplementary Software 1
Description: Custom scripts used for the quantitative analysis of live cell imaging
