# Supplementary material for: Acyl carrier protein promotes MukBEF action in Escherichia coli chromosome organization-segregation
Source: Nat Commun. 2021 Nov 18;12:6721. doi: 10.1038/s41467-021-27107-9 (PMC8602292; doi:10.1038/s41467-021-27107-9)
Supplement: Supplementary file 5 — Reporting Summary [file 41467_2021_27107_MOESM5_ESM.pdf]

nature research

Corresponding author(s): David J Sherratt

Last updated by author(s): Oct 30, 2021

Reporting Summary

Nature Research wishes to improve the reproducibility of the work that we publish. This form provides structure for consistency and transparency in reporting. For further information on Nature Research policies, see our Editorial Policies and the Editorial Policy Checklist.

Statistics

For all statistical analyses, confirm that the following items are present in the figure legend, table legend, main text, or Methods section.

n/a	Confirmed	
☐	☒	The exact sample size (*n*) for each experimental group/condition, given as a discrete number and unit of measurement
☐	☒	A statement on whether measurements were taken from distinct samples or whether the same sample was measured repeatedly
☐	☒	The statistical test(s) used AND whether they are one- or two-sided<br>*Only common tests should be described solely by name; describe more complex techniques in the Methods section.*
☒	☐	A description of all covariates tested
☒	☐	A description of any assumptions or corrections, such as tests of normality and adjustment for multiple comparisons
☐	☒	A full description of the statistical parameters including central tendency (e.g. means) or other basic estimates (e.g. regression coefficient) AND variation (e.g. standard deviation) or associated estimates of uncertainty (e.g. confidence intervals)
☒	☐	For null hypothesis testing, the test statistic (e.g. *F*, *t*, *r*) with confidence intervals, effect sizes, degrees of freedom and *P* value noted<br>*Give P values as exact values whenever suitable.*
☒	☐	For Bayesian analysis, information on the choice of priors and Markov chain Monte Carlo settings
☒	☐	For hierarchical and complex designs, identification of the appropriate level for tests and full reporting of outcomes
☒	☐	Estimates of effect sizes (e.g. Cohen's *d*, Pearson's *r*), indicating how they were calculated

Our web collection on statistics for biologists contains articles on many of the points above.

Software and code

Policy information about availability of computer code

Data collection	ATP hydrolysis assays were measured using a BMG Labtech PherAstar FS plate reader.<br>Native-state ESI-MS spectrometry were collected using a Q-Exactive UHMR mass spectrometer (ThermoFisher).<br>Proteomic MS data were acquired in the Orbitrap (Scan range 350-1500m/z, resolution 70,000; AGC target, 3e6, maximum injection time, 50ms). Peptides were identified using pLink software version 2.3.9.<br>Epifluorescence images were acquired on a Nikon Ti-E inverted microscope equipped with a perfect focus system, a 100× NA 1.4 oil immersion objective (Nikon), an sCMOS camera (Hamamatsu Flash 4), a motorized stage (Nikon), an LED excitation source (Lumencor SpectraX) and a temperature chamber (Okolabs). Images were acquired using NIS-Elements software (Nikon)
Data analysis	ATP hydrolysis assays were analyzed using MARS data analysis software version 2.10, data was then plotted in GraphPad Prism version 8.3.0.<br>Native-state ESI-MS spectrometry were analyzed using Xcalibur version 4.2 and UniDec.<br>Tandem MS/MS data of cross-linked peptides were extracted and annotated pLabel version 2.4.<br>Cell segmentation and spot detection from the fluorescence channel were performed using SuperSegger.<br>The percentages of cells containing one or more spots, distances to the closest ori1/ter3 marker, localization along the long cell axis and percentages of anucleate cell formation were calculated using a custom script in MATLAB (MathWorks).

For manuscripts utilizing custom algorithms or software that are central to the research but not yet described in published literature, software must be made available to editors and reviewers. We strongly encourage code deposition in a community repository (e.g. GitHub). See the Nature Research guidelines for submitting code & software for further information.

Data

Policy information about availability of data

All manuscripts must include a data availability statement. This statement should provide the following information, where applicable:

- Accession codes, unique identifiers, or web links for publicly available datasets
- A list of figures that have associated raw data
- A description of any restrictions on data availability

The mass spectrometry proteomics data generated in this study have been deposited in the ProteomeXchange Consortium via the PRIDE57 partner repository database with the dataset identifiers PXD026017 and PXD026062. Source data are provided with this paper. All other raw data and code that supports the findings of this study are available from the corresponding author upon reasonable request.

Field-specific reporting

Please select the one below that is the best fit for your research. If you are not sure, read the appropriate sections before making your selection.

- [x] Life sciences
- [ ] Behavioural & social sciences
- [ ] Ecological, evolutionary & environmental sciences

For a reference copy of the document with all sections, see nature.com/documents/nr-reporting-summary-flat.pdf

Life sciences study design

All studies must disclose on these points even when the disclosure is negative.

Sample size	No statistical methods were used to determine sample size. The sample size used are standard within the field and described within the methods an/or figure legends.
Data exclusions	No data points were excluded from the analysis
Replication	Multiple independent experiments were carried out and all attempts to reproduce data were successful. The number of independent experiments are listed in the figure legends.
Randomization	Microscopy experiments were carried out on three randomly chosen E. coli transformants with the same genetic background.
Blinding	Experiments were performed comparing various protein complexes and mutations; it was necessary for the researchers to be aware of sample composition. All experimental methods and analysis were applied equally to all samples and controls.

Reporting for specific materials, systems and methods

We require information from authors about some types of materials, experimental systems and methods used in many studies. Here, indicate whether each material, system or method listed is relevant to your study. If you are not sure if a list item applies to your research, read the appropriate section before selecting a response.

Materials & experimental systems

n/a	Involved in the study	
[ ]	[x]	Antibodies
[x]	[ ]	Eukaryotic cell lines
[x]	[ ]	Palaeontology and archaeology
[x]	[ ]	Animals and other organisms
[x]	[ ]	Human research participants
[x]	[ ]	Clinical data
[x]	[ ]	Dual use research of concern

Methods

n/a	Involved in the study	
[x]	[ ]	ChIP-seq
[x]	[ ]	Flow cytometry
[x]	[ ]	MRI-based neuroimaging

Antibodies

Antibodies used	anti-AcpP (LSBio, LS-C370023, 1:5000 dilution) as primary and goat anti-rabbit HPR (ThermoFisher, 65-6120, 1:10000 dilution) as secondary antibody.
Validation	The commercial anti-AcpP antibody against AcpP has been validated for use in ELISA by the company. The antibody was verified against recombinant and native E.coli AcpP via western blotting.
